# Treatment of Severe Acute Pancreatitis and Related Lung Injury by Targeting Gasdermin D-Mediated Pyroptosis

**DOI:** 10.3389/fcell.2021.780142

**Published:** 2021-11-11

**Authors:** Jinxiang Wu, Jintao Zhang, Jiping Zhao, Shihong Chen, Tao Zhou, Jianwei Xu

**Affiliations:** ^1^Department of Pulmonary and Critical Care Medicine, Qilu Hospital, Cheeloo College of Medicine, Shandong University, Jinan, China; ^2^Department of Respiratory, Shandong Qianfoshan Hospital, Cheeloo College of Medicine, Shandong University, Jinan, China; ^3^Department of Pancreatic Surgery, General Surgery, Qilu Hospital, Cheeloo College of Medicine, Shandong University, Jinan, China; ^4^Department of Gastroenterology, Qilu Hospital, Cheeloo College of Medicine, Shandong University, Jinan, China

**Keywords:** severe acute pancreatitis, acute lung injury, pyroptosis, GSDMD, disulfiram, programmed cell death, inflammation

## Abstract

The functional relevance and effects of the pyroptosis executioner gasdermin D (GSDMD) on severe acute pancreatitis (SAP)-associated lung injury are unclear. We established caerulein-induced mouse models of SAP-associated lung injury, which showed that GSDMD-mediated pyroptosis was activated in both pancreatic and lung tissues. Compared with *Gsdmd* wild-type SAP mouse models, *Gsdmd* knockout (*Gsdmd^–/–^*) ameliorated SAP-induced pancreas and related lung injury. Additionally, we investigated the effects of disulfiram on the treatment of SAP. Disulfiram is a Food and Drug Administration (FDA)-approved anti-alcoholism drug, which is reported as an effective pyroptosis inhibitor by either directly covalently modifying GSDMD or indirectly inhibiting the cleavage of GSDMD via inactivating Nod-like receptor protein 3 inflammasome. We demonstrated that disulfiram inhibited the cleavage of GSDMD, alleviated caerulein-induced SAP and related lung injury, and decreased the expression levels of proinflammatory cytokines (IL-1β and IL-18). Collectively, these findings disclosed the role of GSDMD-mediated pyroptosis in SAP and the potential application of disulfiram in the treatment of SAP.

## Introduction

Acute pancreatitis (AP) is an inflammatory disorder featured by the destruction of acinar cells. Mostly, AP is mild and self-limited. However, approximately over 20% of all patients with AP will develop to be a severe condition with multiorgan dysfunction, such as the lungs, kidneys, heart, and gut (Leppäniemi et al., 2019; Nassar and Qunibi, 2019), which results in a mortality of 15% (van Santvoort et al., 2011). Acute lung injury (ALI) is the most common distant organ dysfunction secondary to severe AP (SAP) with an incidence of 27.7% (Lei et al., 2013; Barreto et al., 2021), which accounts for up to 60% SAP-associated deaths (Elder et al., 2012). One goal of AP treatment is reducing the incidence of mild AP developing to SAP and the mortality of SAP. Understanding the mechanisms of SAP-associated ALI is helpful to effective treatment of this severe disease.

Pyroptosis is the inflammasome-induced programmed cell death mediated by gasdermins (Shi et al., 2015; Broz et al., 2020), which is featured by cell membrane pore formation, cytoplasmic swelling, membrane rupture, and the release of cytosolic contents (such as IL-1β and IL-18), amplifying the local or systemic inflammatory effects (Wang et al., 2019; Broz et al., 2020). Pyroptosis or gasdermins are involved in several diseases, including viral or bacterial infections, autoinflammatory genetic diseases, complex diseases exacerbated by inflammation, and cancer; inhibiting pyroptosis or gasdermins is a promising strategy to intervene in these diseases (Liu et al., 2021). However, only very few studies focused on the role of pyroptosis in SAP-associated ALI (Wu et al., 2020; Gao et al., 2021; Xu et al., 2021). It is unclear whether the targeted therapy of pyroptosis will be effective.

This study aimed to disclose the role of pyroptosis in SAP and related ALI in mouse models, and both wild-type (WT) and gasdermin D (*Gsdmd*, an import member of the gasdermin family) knockout mice were used. Additionally, the therapeutic effect of disulfiram [DSF, a Food and Drug Administration (FDA)-approved anti-alcoholism drug, is an effective pyroptosis inhibitor by either directly covalently modifying GSDMD or indirectly inhibiting the cleavage of GSDMD via inactivating Nod-like receptor protein 3 (NLRP3) inflammasome (Deng et al., 2020; Hu et al., 2020; Zhang et al., 2021)] was evaluated.

## Materials and Methods

### Ethics Statement

This study was approved by the Medical Ethics Committee of Qilu Hospital of Shandong University.

### Animal Models

C57BL/6 WT mice with a weight of 20 ± 2 g and age of 6 weeks were purchased from Beijing Vital River Laboratory Animal Technology Co., Ltd. (Beijing, China). *Gsdmd* knockout (*Gsdmd*^–/–^) mice were a gift from Prof. Feng Shao (National Institute of Biological Sciences, Beijing, China) (Shi et al., 2015).

To investigate the role of GSDMD-mediated pyroptosis in SAP, male WT and *Gsdmd* ^–/–^ mice were randomly assigned to the following four groups: (1) WT-sham group, 0.9% saline, intraperitoneal (i.p.) injection; (2) WT-SAP group; (3) *Gsdmd* ^–/–^ sham group; and (4) *Gsdmd* ^–/–^ SAP group. The SAP model was established according to previous studies (Wang et al., 2014; Yang et al., 2019; Liang et al., 2020). In brief, caerulein (cat. no. C9026, Sigma, St. Louis, MO, United States) (50 μg/kg) was intraperitoneally injected at 1-h interval for seven times, and with the last injection of caerulein, the mice received an injection of lipopolysaccharide (LPS; cat. no. L2630, Sigma) (10 mg/kg, i.p.). The timeframe of caerulein and LPS injection is displayed in Supplementary Figure 1.

To investigate the effects of DSF (MedChemExpress, Princeton, NJ, United States; no. HY-B0240) on the treatment of SAP, male WT mice were randomly assigned to the following six groups: (1) the sham group, in which the mice received normal saline (0.9%, i.p.); (2) DSF group (50 mg/kg, i.p.); (3) the SAP group; (4) SAP + DSF (25 mg/kg, i.p.); (5) SAP + DSF (50 mg/kg, i.p.); and (6) SAP + DSF (100 mg/kg, i.p.). Briefly, mice were pretreated with DSF (25 mg/kg, i.p.; 50 mg/kg, i.p.; and 100 mg/kg, i.p.) 24 h before injection of caerulein, and 24 and 4 h before injection of LPS, retrospectively. The timeframe of DSF injection is displayed in Supplementary Figure 1.

All animals were anesthetized via i.p. injection of pentobarbital sodium (Seebio, Shanghai, China; P3761) (50 mg/kg) and then killed 24 h after the last injection.

### Sample Collection

Blood samples were collected by eye enucleation. After centrifugation, the serum was collected and stored at −80°C for further ELISA analysis. The pancreases and left lungs were enucleated. One part was frozen at −80°C, and the other part was fixed in paraformaldehyde and then embedded in paraffin.

The right lung was enucleated, the surface water was absorbed with a filter paper, and then the weight was measured and recorded as the wet weight. After that, the lung was then dried at 70°C in an oven for 48 h, and the dry weight was measured. The wet/dry ratio was calculated.

### Enzyme-Linked Immunosorbent Assay

Serum amylase (cat. no. ab193692, Abcam, Cambridge, United Kingdom), lipase (cat. no. ab102524, Abcam), IL-1β (cat. no. ab197742, Abcam), IL-18 (cat. no. ab216165, Abcam), IL-6 (cat. no. ab222503, Abcam), and tumor necrosis factor-alpha (TNF-α) (cat. no. ab208348, Abcam) were detected using ELISA kits according to the manufacturer’s instructions.

### Histological Examination and Organ Injure Score

Paraffin-embedded slides of the pancreatic and lung tissues were stained with H&E and then observed under a light microscope. The pancreatic pathology was evaluated according to Schmidt’s criteria (Schmidt et al., 1992). The lung pathology was evaluated according to Mayer’s criteria (Mayer et al., 1999).

### Immunohistochemistry

The slides were deparaffinized in xylene and rehydrated in a graded ethanol series. Endogenous peroxidase activity was blocked with 3% H_2_O_2_ for 10 min. Antigen retrieval was performed via incubating the slides in 0.1% trypsin and heating them in a microwave oven. The slides were incubated with primary antibodies (1:200 dilution) at 4°C overnight, with the primary antibodies including anti-IL-1β (cat. no. ab9722, Abcam) (1:200) and anti-IL-18 (cat. no. ab71495, Abcam) (1:200). Then, the slides were incubated with horseradish peroxidase (HRP)-conjugated secondary antibody (1:200) for 30 min at 37°C. Diaminobenzidine (DAB) used as a chromogen. The slides were counterstained with hematoxylin.

### Immunofluorescence Assay

The slides were prepared and covered with 1% bovine serum albumin in phosphate-buffered saline (PBS). Slides were incubated overnight at 4°C with primary antibodies to IL-1β (1:300) and IL-18 (1:300) and then washed and incubated with the secondary antibody for 2 h at room temperature in the dark. After washing, the slides were stained with 4′,6-diamidino-2-phenylindole (DAPI) (Sigma-Aldrich, St. Louis, MO, United States) and visualized with a fluorescence microscope.

### Western Blotting

Total protein was isolated from the pancreatic and lung tissues. The concentrations were measured by bicinchoninic acid assay. Protein samples were loaded and separated by sodium dodecyl sulfate–polyacrylamide gel electrophoresis and then transferred to a polyvinylidene difluoride membrane (Millipore, Billerica, MA, United States). The membrane was blocked with 5% skimmed milk at room temperature for 1 h and then incubated with primary antibodies to IL-1β (1:1,000), IL-18 (1:1,000), and GSDMD (cat. no. ab210070, Abcam) (1:1,000) overnight at 4°C. The membrane was washed and then incubated with secondary antibodies (1:2,000) (Applygen, Beijing, China) at room temperature for 1 h. Finally, the protein bands were scanned with echochemiluminescence detection system and quantified using Image-Pro Plus 6.0 software (Media Cybernetics, Rockville, MD, United States).

### Statistics

Data were collected and analyzed by GraphPad Prism 7.0 software and presented as the means ± SD. The statistics were analyzed by using two-tailed unpaired *t*-test for two groups or two-way ANOVA for multiple groups. *p*-values were provided as ^∗^*p* < 0.05 and *^#^p* < 0.05.

## Results

### Activation of Gasdermin D-Mediated Pyroptosis in Pancreatic and Lung Tissues in Severe Acute Pancreatitis Models

**FIGURE 1:**
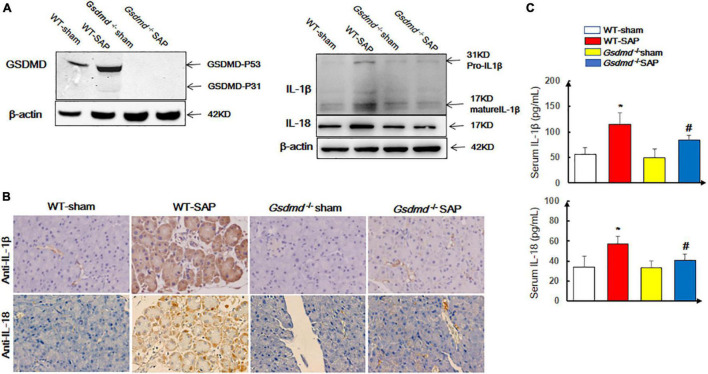
GSDMD-mediated pyroptosis is activated in pancreatic tissues in severe acute pancreatitis mouse models. Mice were sacrificed 24 h after LPS injection, and samples were collected. **(A,B)** The pyroptosis-associated molecules GSDMD, IL-1β, and IL-18 were analyzed by Western blotting and IHC. Scale bar, ×200. **(C)** Serum IL-1β and IL-18 levels were detected by ELISA. *p < 0.05 vs. other groups; *^#^p* < 0.05 vs. *Gsdmd* ^–/–^ sham group. Experiments were repeated three times. Data are expressed as mean ± SD, *n* = 10 per WT group, *n* = 8 per *Gsdmd* ^–/–^ group. GSDMD, gasdermin D; LPS, lipopolysaccharide; IHC, immunohistochemistry; WT, wild type.

We first examined the status of pyroptosis in SAP mouse models. The data showed that the expression levels of GSDMD were significantly upregulated in the pancreatic tissue (Figure 1A) in the WT-SAP group. The levels of proinflammatory cytokines (IL-1β and IL-18) in both pancreatic tissues and serum in the WT-SAP group were significantly increased (Figures 1A–C). *Gsdmd* knockout decreased the levels of IL-1β and IL-18 in pancreatic tissues and serum (Figure 1).

**FIGURE 2:**
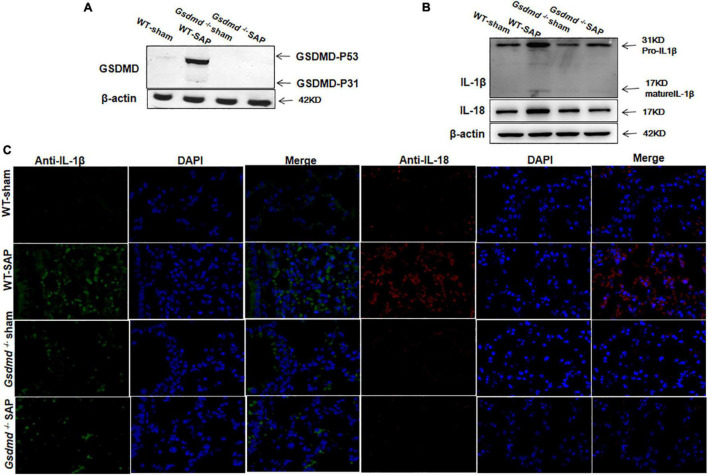
GSDMD-mediated pyroptosis is activated in lung tissues in severe acute pancreatitis mouse models. Mice were sacrificed 24 h after LPS injection, and samples were collected. **(A,B)** The pyroptosis-associated molecules GSDMD, IL-1β, and IL-18 were analyzed in the lung by Western blotting. Experiments were repeated three times. **(C)** Immunofluorescence labeling of IL-1β (green), IL-18 (red), and DAPI (blue) in the four groups, Scale bar, × 200. All the data are expressed as mean ± SD, *n* = 10 per WT group, *n* = 8 per *Gsdmd* ^–/–^ group. GSDMD, gasdermin D; LPS, lipopolysaccharide; WT, wild type.

Similarly, increases of GSDMD, IL-1β, and IL-18 were observed in lung tissues in WT-SAP mouse models, while *Gsdmd* knockout decreased the levels of IL-1β and IL-18 in lung tissues (Figure 2).

### *Gsdmd* Knockout Ameliorated Severe Acute Pancreatitis-Induced Pancreas and Related Lung Injury

**FIGURE 3:**
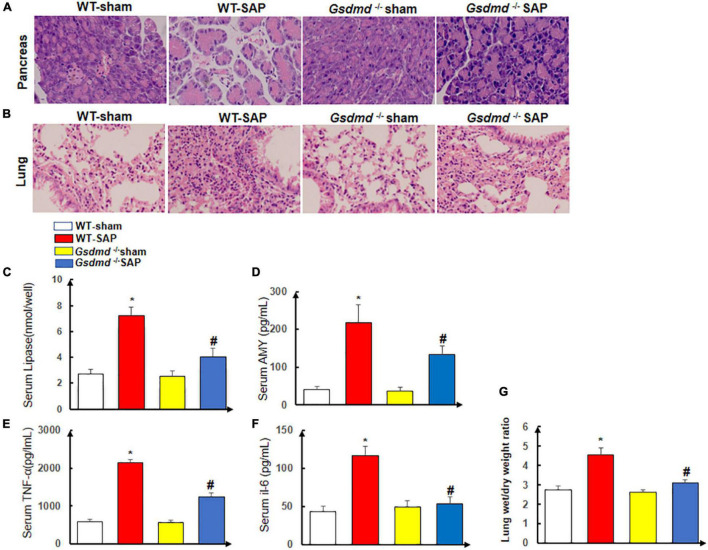
*Gsdmd* deficiency attenuates the injury of the pancreas and lung. Mice were sacrificed 24 h after LPS injection, and samples were collected. **(A,B)** H&E staining of the pancreas and lung in four groups was performed, and the morphologies were photographed at ×200 enlargement. **(C–F)** The levels of serum lipase, amylase, TNF-α, and IL-6. Experiments were repeated three times. *p < 0.05 vs. other groups; *^#^p* < 0.05 vs. *Gsdmd* ^–/–^ sham group in the expression of lipase, amylase, TNF-α, and IL-6. **(G)** Lung tissue wet/dry ratio was measured. All the data are expressed as mean ± SD, *n* = 10 per WT group, *n* = 8 per *Gsdmd* ^–/–^ group. LPS, lipopolysaccharide; WT, wild type.

There were no histological changes of the pancreases with regularly arranged acini in the WT-sham group. In the WT-SAP group, edema and disordered arrangement of acini with destruction, edema and congestion of interstitial components, and inflammatory cell infiltration could be observed. The injuries of the pancreases in the *Gsdmd*^–/–^ SAP group were significantly attenuated by both gross and histological observations when compared with those in the WT-SAP group (Figure 3A).

The lungs in the WT-sham group were normal without edema and congestion, yet edema and bleeding under the pulmonary capsule were observed in the WT-SAP group; the inflammation of the lung in the *Gsdmd* ^–/–^ SAP group was significantly lower than that in the WT-sham group (Figure 3B).

The levels of circulating AMY, lipase, amylase, TNF-α, and IL-6 in the WT-SAP group were significantly higher than those in the WT-sham, *Gsdmd* ^–/–^ sham, and *Gsdmd* ^–/–^ SAP groups. *Gsdmd* knockout had lower levels of circulating AMY, lipase, amylase, TNF-α, and IL-6 than the WT-SAP group (Figures 3C–F). *Gsdmd* deficiency also significantly diminished SAP-induced increases in lung wet/dry ratio (Figure 3G).

### Disulfiram Ameliorated the Injury of the Pancreas and Lung in Severe Acute Pancreatitis Model

As known, DSF is an effective pyroptosis inhibitor that directly covalently modifies GSDMD or indirectly inhibits the cleavage of GSDMD via inactivating NLRP3 inflammasome (Deng et al., 2020; Hu et al., 2020; Zhang et al., 2021). In consideration of the anti-pyroptosis effects of DSF by regulating GSDMD, the therapeutic effect of DSF would not be obviously demonstrated in *Gsdmd*^–/–^ mouse models; we investigated the therapeutic effects of DSF based on WT mouse models.

**FIGURE 4:**
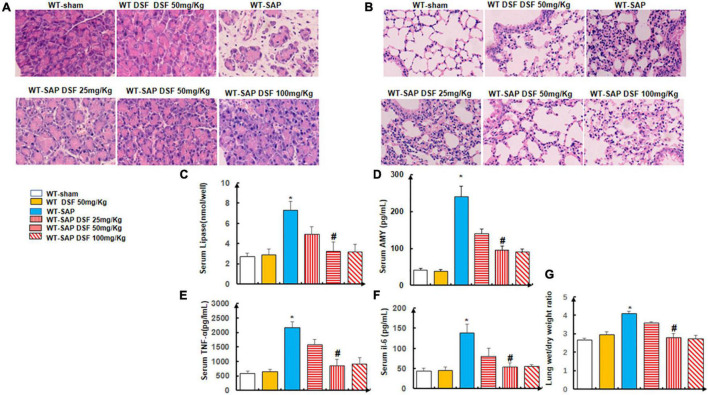
Inhibition of pyroptosis with DSF alleviates the inflammation of the pancreas and lung. WT mice were separately treated with DSF of 25, 50, and 100 mg/kg. At 24 h after LPS injection, all mice were sacrificed. **(A,B)** Representative H&E staining of pancreatic and lung tissue. Scale bars, × 200. **(C–F)** Levels of lipase, amylase, TNF-α, and IL-6 in the serum were tested using ELISA. Experiments were repeated three times. **(G)** Wet/dry ratio was measured in the lung tissue. Data are presented as mean ± SD (*n* = 10 per group). *p < 0.05 vs. other groups; *^#^p* < 0.05 vs. WT-SAP group, WT-SAP DSF (25 mg/kg); *^#^p* > 0.05 vs. sham, sham DSF (50 mg/kg), WT-SAP DSF (100 mg/kg). DSF, disulfiram; WT, wild type; LPS, lipopolysaccharide; SAP, severe acute pancreatitis.

We showed that DSF treatment alleviated the injuries of the pancreases and lungs by both gross and histological observation in the WT-SAP group when compared with the sham group (Figures 4A,B), which also decreased the levels of circulating AMY, lipase, amylase, TNF-α, and IL-6 as shown in Figures 4C–G.

### Disulfiram Inhibited the Cleavage of Gasdermin D and Decreased the Levels of IL-1β and IL-18

**FIGURE 5:**
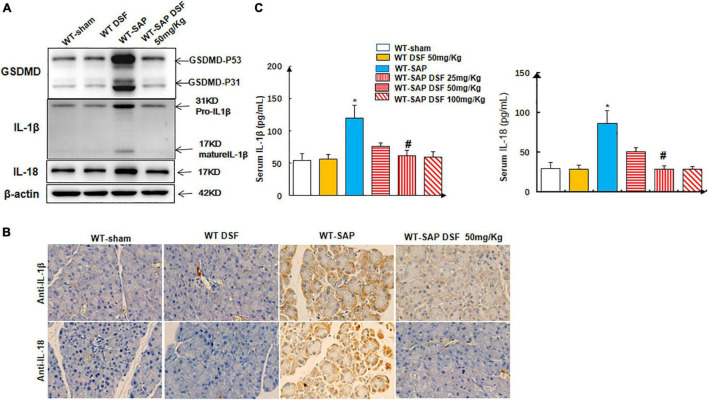
DSF inhibits the cleavage of GSDMD and decreases the levels of IL-1β and IL-18 in pancreatic tissues. WT-sham and WT-SAP mouse models were treated with DSF (50 mg/kg). The mice were sacrificed 24 h after LPS injection. **(A,B)** Western blotting and IHC analysis of GSDMD, IL-1β, and IL-18 in pancreatic tissue. Scale bars, ×200. **(C)** Serum IL-1β and IL-18 were analyzed by ELISA. Experiments were repeated three times. Data are presented as mean ± SD (*n* = 10 per group). *p < 0.05 vs. other groups; *^#^p* < 0.05 vs. WT-SAP group, WT-SAP DSF (25 mg/kg); *^#^p* > 0.05 vs. sham + sham DSF (50 mg/kg), WT-SAP DSF (100 mg/kg). DSF, disulfiram; GSDMD, gasdermin D; WT, wild type; SAP, severe acute pancreatitis; LPS, lipopolysaccharide; IHC, immunohistochemistry.

Treatment with DSF significantly decreased the levels of cleaved GSDMD in pancreatic tissues compared with the untreated group in WT-SAP models. Furthermore, WT-SAP mice undergoing DSF treatment presented lower levels of IL-1β and IL-18 in both pancreatic tissues and serum than the untreated mice (Figure 5).

**FIGURE 6:**
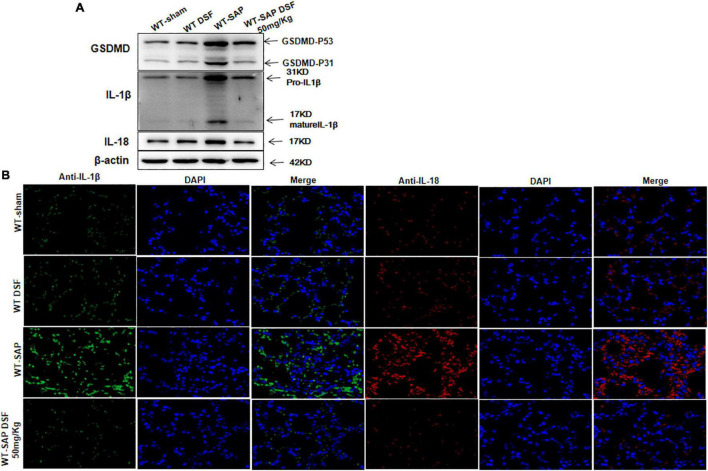
DSF inhibits the cleavage of GSDMD and decreases the levels of IL-1β and IL-18 in lung tissues. WT-sham and WT-SAP mouse models were treated with DSF (50 mg/kg). The mice were sacrificed 24 h after LPS injection. **(A)** Western blotting analysis of GSDMD, IL-1β, and IL-18 in the lung. Scale bars, × 200. Experiments were repeated three times. **(B)** Immunofluorescence labeling of IL-1β (green), IL-18 (red), and DAPI (blue) in the four WT groups, Scale bar, × 200. Data are presented as mean ± SD (*n* = 10 per group). DSF, disulfiram; GSDMD, gasdermin D; WT, wild type; SAP, severe acute pancreatitis; LPS, lipopolysaccharide.

Similarly, DSF inhibited the cleavage of GSDMD and decreased the levels of IL-1β and IL-18 levels in lung tissues in WT-SAP models (Figure 6).

## Discussion

SAP is an inflammatory disease featured by the activation of trypsinogen, infiltration of inflammatory cells, and destruction of acinar cells. The local inflammation of the pancreas results in the proinflammatory cytokines storm, which will induce systemic inflammation and multiple organ dysfunction. Blocking the cascaded amplification of the inflammation can improve the outcomes of SAP. Pyroptosis is an inflammasome-induced programmed cell death (Shi et al., 2015; Broz et al., 2020). Activation of cells pyroptosis induces membrane pore formation, which permits the release of proinflammatory cytokines and then amplifies the local or systemic inflammatory effects (Wang et al., 2019; Broz et al., 2020). Inactivation of the process of pyroptosis can inhibit the production and release of proinflammatory cytokines, which might be a promising strategy to treat inflammatory diseases (Liu et al., 2021). However, the roles of pyroptosis in SAP-associated ALI are still unknown. Our study indicated that GSDMD-mediated pyroptosis involved in the progression of SAP. Both *Gsdmd* knockout and treatment with DSF inhibited GSDMD-mediated pyroptosis, decreased the release of proinflammatory cytokines, and alleviated the injury of the pancreas and lungs in SAP mouse models.

Gasdermins contain six members in humans (including A, B, C, D, E, and DFNB59) and 10 members in mice (A1/2/3, B, C1/2/3/4, D, and DFNB59).^7^ Except DFNB59, all other gasdermins consist of two conserved domains that can be cleaved to C-terminal inhibitory domain and N-terminal effector domain by activating caspase-1 in the canonical pathway (Kovacs and Miao, 2017; Wang et al., 2019). The N-terminal fragment interacts with the cell plasma membrane to form membrane pores, which further promotes the release of inflammatory (including IL-1β and IL-18), cell swelling, and membrane rupture (Kovacs and Miao, 2017).

GSDMD is a 53-kDa protein and is extensively investigated in pyroptosis. Only few studies reported the involvement of GSDMD-mediated pyroptosis in the pathogenesis of AP. Wang et al. (2020) indicated that the circular RNA circHIPK3 induced pyroptosis in pancreatic acinar cells via regulating the miR-193a-5p/GSDMD axis and then aggravated AP. Lin et al. (2021) showed that downregulating GSDMD protein reduced pancreatitis associated with pyroptosis, attenuated the degrees of edema and hemorrhage in the intestinal tract, and alleviated the pathological changes of pancreatic tissue. Recently, an in-depth study reported by Gao et al. (2021) indicated that NLRP3 inflammasome and GSDMD activation-mediated pyroptosis in acinar cells induced pancreatic necrosis and systemic inflammation in AP. Co-application of RIP3 inhibitor on *Gsdmd*^–/–^ mice increased protection on caerulein-induced AP.

Pyroptosis is involved in the progression of SAP-associated ALI. Activation of NLRP3 inflammasome-dependent pyroptosis in alveolar macrophages (AMs) was responsible for the lung injury secondary to SAP. Plasma-derived exosomes from SAP mice were capable of triggering NLRP3-dependent pyroptosis in AMs (Wu et al., 2020). Emodin might inhibit the cold-inducible RNA-binding protein (CIRP)-mediated activation of the NLRP3/IL-1β/CXCL1 signaling in rats, which then ameliorated the SAP-associated ALI (Xu et al., 2021). Our study confirmed the crucial role of GSDMD-mediated pyroptosis in SAP-associated ALI.

DSF is an FDA-approved anti-alcoholism drug. As early as 2008, Kast proposed the potential therapeutic value of DSF on AP based on the relationship between IL-18 and AP (Kast, 2008), yet our study was the first report to confirm this effect via animal experiments. The anti-inflammation effects of DSF have also been reported in other diseases, including obesity and its metabolic comorbidities (Bernier et al., 2020), non-alcoholic fatty liver disease (NAFLD) (Liu et al., 2018), LPS-induced septic death (Hu et al., 2020), LPS-induced peritoneal inflammation, and monosodium urate (MSU) crystals that induced gouty inflammation (Deng et al., 2020). The mechanisms of DSF on the treatment of inflammation diseases are unclear. DSF could modulate lipid metabolism and oxidative stress in mouse models of NAFLD (Liu et al., 2018), as well as could inactivate the NLRP3 inflammasome and reduce mitochondrial-independent reactive oxygen species (ROS) production (Deng et al., 2020). Recently, some studies demonstrated the effects of DSF on pyroptosis. Zhang et al. (2021) showed that DSF inhibited the cleavage of GSDMD in HK-2 cells without preventing inflammation if the cells had already undergone pyroptosis. Hu et al. (2020) indicated that DSF blocked pyroptosis and cytokine release in cells and LPS-induced septic death in mice via covalently modifying Cys191/Cys192 in GSDMD to block pore formation, but still allowed IL-1β and GSDMD processing. In consideration of the crucial role of GSDMD-mediated pyroptosis in inflammation diseases, Schmidt and Latz (2020) pointed out the role of “jack of all trades” of DSF in inhibiting inflammation when they commented on Hu’s publication (Hu et al., 2020). Our study was an important evidence of this speculation. DSF not only alleviated the injury of the pancreas but also protected the lung, which meant that treatment of DSF could prevent the disease from being mild to becoming severe, reduce the proportion of patients with severe conditions, and reduce mortality. It is worth looking forward to further conduct clinical trials.

In conclusion, we reported on GSDMD-mediated pyroptosis involved in the progression of SAP-associated ALI. Targeted therapy of GSDMD using DSF inhibited pyroptosis and alleviated the injury of the pancreas and lung.

## Data Availability Statement

The original contributions presented in the study are included in the article/Supplementary Material, further inquiries can be directed to the corresponding author/s.

## Ethics Statement

The animal study was reviewed and approved by Medical Ethics Committee of Qilu Hospital of Shandong University.

## Author Contributions

JX performed the study concept and design. JW and JX performed the development of methodology and the writing, review, and revision of the manuscript. JW, JTZ, and JPZ provided the analysis, interpretation of data, and the statistical analysis. JX, JW, JTZ, SC, and TZ provided the technical and material support. All authors read and approved the final manuscript.

## Conflict of Interest

The authors declare that the research was conducted in the absence of any commercial or financial relationships that could be construed as a potential conflict of interest.

## Publisher’s Note

All claims expressed in this article are solely those of the authors and do not necessarily represent those of their affiliated organizations, or those of the publisher, the editors and the reviewers. Any product that may be evaluated in this article, or claim that may be made by its manufacturer, is not guaranteed or endorsed by the publisher.

## Supplementary Material

Supplementary Figure 1 **(A)** The timeframe of caerulein and LPS injections. **(B)** The timeframe of caerulein, LPS, and DSF injections.
