# Supplementary figures and images for: Treatment of Severe Acute Pancreatitis and Related Lung Injury by Targeting Gasdermin D-Mediated Pyroptosis
Source: Front Cell Dev Biol. 2021 Nov 11;9:780142. doi: 10.3389/fcell.2021.780142 (PMC8632453; doi:10.3389/fcell.2021.780142)

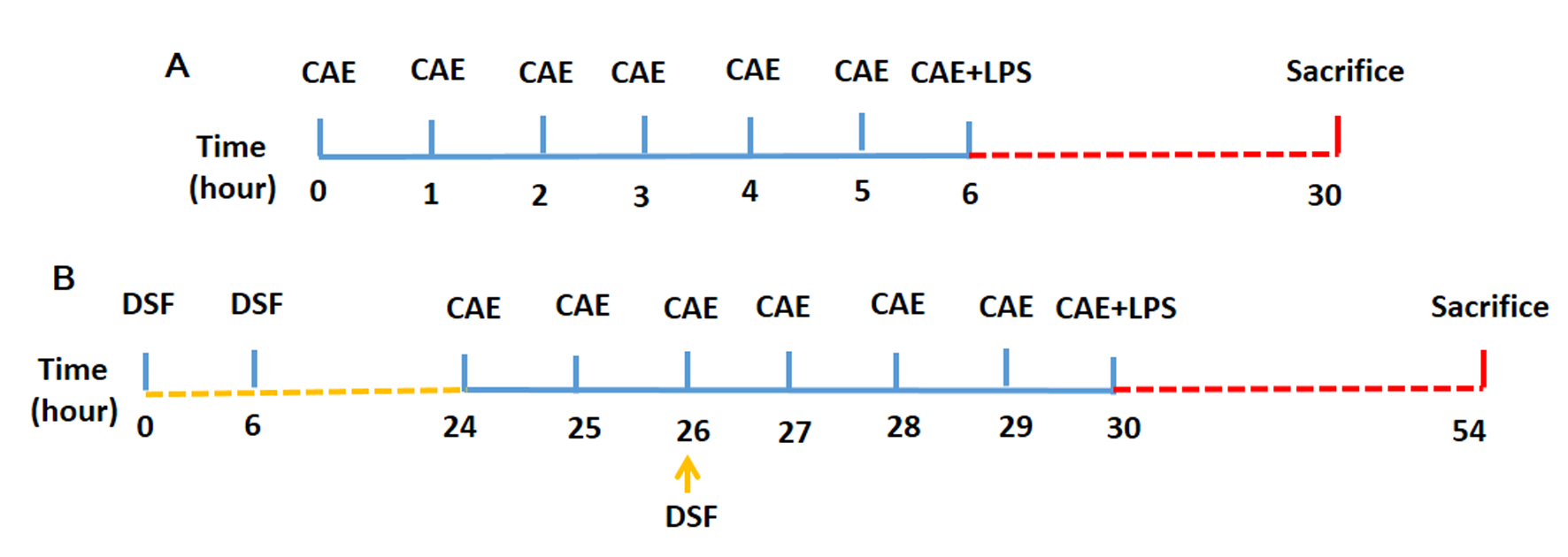

Supplement: Supplementary Figure 1 — (A) The timeframe of caerulein and LPS injections. (B) The timeframe of caerulein, LPS, and DSF injections. [file Image_1.TIF]
